# Identifying the Determinants of Distribution of *Oncomelania hupensis* Based on Geographically and Temporally Weighted Regression Model along the Yangtze River in China

**DOI:** 10.3390/pathogens11090970

**Published:** 2022-08-25

**Authors:** Zhe Wang, Lu Liu, Liang Shi, Xinyao Wang, Jianfeng Zhang, Wei Li, Kun Yang

**Affiliations:** 1 School of Public Health, Nanjing Medical University, Nanjing 211166, China; 2 Key Laboratory of National Health and Family Planning Commission on Parasitic Disease Control and Prevention, Jiangsu Provincial Key Laboratory on Parasite and Vector Control Technology, Jiangsu Institute of Parasitic Diseases, Wuxi 214064, China; 3 Public Health Research Center, Jiangnan University, Wuxi 214122, China

**Keywords:** *Oncomelania hupensis*, heterogeneity, spatial autocorrelation, geographical and temporal weighted regression, Yangtze River

## Abstract

Background: As the unique intermediate host of *Schistosoma japonicum*, the geographical distribution of *Oncomelania hupensis (O. hupensis)* is an important index in the schistosomiasis surveillance system. This study comprehensively analyzed the pattern of snail distribution along the Yangtze River in Jiangsu Province and identified the dynamic determinants of the distribution of *O. hupensis*. Methods: Snail data from 2017 to 2021 in three cities (Nanjing, Zhenjiang, and Yangzhou) along the Yangtze River were obtained from the annual cross-sectional survey produced by the Jiangsu Institute of Parasitic Diseases. Spatial autocorrelation and hot-spot analysis were implemented to detect the spatio–temporal dynamics of *O. hupensis* distribution. Furthermore, 12 factors were used as independent variables to construct an ordinary least squares (OLS) model, a geographically weighted regression (GWR) model, and a geographically and temporally weighted regression (GTWR) model to identify the determinants of the distribution of *O. hupensis*. The adjusted coefficients of determination (adjusted R^2^, AICc, RSS) were used to evaluate the performance of the models. Results: In general, the distribution of *O. hupensis* had significant spatial aggregation in the past five years, and the density of *O. hupensis* increased eastwards in the Jiangsu section of the lower reaches of the Yangtze River. Relatively speaking, the distribution of *O. hupensis* wase spatially clustered from 2017 to 2021, that is, it was found that the border between Yangzhou and Zhenjiang was the high density agglomeration area of *O. hupensis* snails. According to the GTWR model, the density of *O. hupensis* was related to the normalized difference vegetation index, wetness, dryness, land surface temperature, elevation, slope, and distance to nearest river, which had a good explanatory power for the snail data in Yangzhou City (adjusted R^2^ = 0.7039, AICc = 29.10, RSS = 6.81). Conclusions: The distribution of *O. hupensis* and the environmental factors in the Jiangsu section of the lower reaches of the Yangtze River had significant spatial aggregation. In different areas, the determinants affecting the distribution of *O. hupensis* were different, which could provide a scientific basis for precise prevention and control of *O. hupensis*. A GTWR model was prepared and used to identify the dynamic determinants for the distribution of *O. hupensis* and contribute to the national programs of control of schistosomiasis and other snail-borne diseases.

## 1. Introduction

Schistosomiasis, a water-borne parasitic disease that results from infection by trematode worms of the genus *Schistosoma*, is prevalent in 78 tropical and sub-tropical countries worldwide. According to the World Health Organization (WHO), it affects more than 230 million people, with an estimated 700 million at risk [1]. In 2020, WHO published a new “road map” targeting the elimination of schistosomiasis by 2030, but continued actions are required to reach this target [2]. In the People’s Republic of China, Schistosomiasis japonica, caused by *S. japonicum*, brought disability and death to millions of people, and had long been an important public health problem before the implementation of its national control plan [3]. After more than 70 years of effort, seven out of twelve endemic provinces reached the criteria of transmission interruption [4]. Jiangsu Province is one of the seven provinces which had met the criteria of transmission interruption by 2019. In Jiangsu Province, more than 90% of schistosomiasis endemic areas are located in marshland and lake regions distributed along the Yangtze River [5]. Nanjing, Zhenjiang, and Yangzhou accounted for 72% of the intermediate host area in Jiangsu Province, and the annual number of monitored cases in these three cities exceeded 60% of the province in total [6].

It is well known that *Oncomelania hupensis* (*O. hupensis*) is the unique intermediate host of *S. japonicum*, whose geographical distribution correlates with that of schistosomiasis [7]. In addition, the distribution of *O. hupensis* is closely related to climate and geographical factors, especially temperature, humidity, altitude, and vegetation coverage [8,9,10]. Some researchers have found that the distribution patterns of *O. hupensis* could be predicted by some environmental factors, such as land surface temperature (LST) and normalized differential vegetation index (NDVI) [11,12]. Regression models have been widely used in studying the ecology of diseases, such as the spatial–temporal variance of distribution of *O. hupensis* and its driving factors [13,14]. Geographically weighted regression (GWR) is one such regression model, which could be used to predict the results of unknown points by establishing a local regression equation of each point in the spatial range to explore the spatial change of the object in a certain scale and the relevant driving factors [15,16,17]. Taking into account the influence of time dimension on diseases, the geographically and temporally weighted regression (GTWR) model integrates time and space dimensions, and has gradually been applied in the study of the spatial and temporal distribution of diseases and the analysis of relevant influencing factors [18,19,20,21].

Due to human mobility, floods, and other factors [22,23], the risk of schistosomiasis recurrence remains a potential threat and deserves unwavering attention. In recent years, with the proposal of the Yangtze River protection policy, the ecological environment around the river has gradually changed, day by day. For instance, because of the rising water level of the Yangtze River, over time, vegetation gradually grew in the demolished factory spaces along the river, which promoted snail propagation and diffusion; and restoration of the wetlands created conditions for snail breeding, and may even create a new snail source [24,25]. As a consequence, the change of geographical environment brings uncertainty to the recurrence of *Oncomelania* snails. Under these circumstances, it is urgent to further explore the spatio–temporal distribution of *O. hupensis* along the Jiangsu section of the Yangtze River and monitor the different determinants from recent years.

This study aimed to explore the spatio–temporal patterns of snail distribution and identify the dynamic determinants of the distribution of *O. hupensis* by the GTWR model along the Yangtze River in Jiangsu Province, so as to contribute to the national programs of control of schistosomiasis and other snail-borne diseases.

## 2. Materials and Methods

### 2.1. Study Area

**Figure 1:**
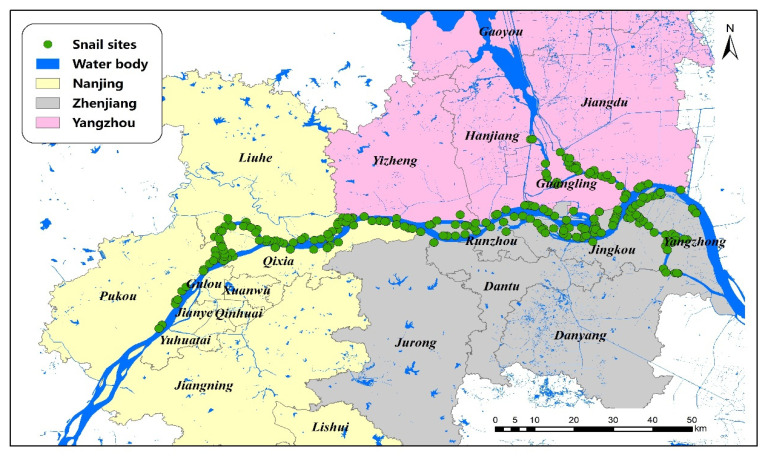
A map of the study area and the sites of *O. hupensis* along the Yangtze River in Jiangsu Province.

The marshland endemic regions of three cities (Nanjing, Zhenjiang and Yangzhou) along the Yangtze River were studied, which are all located in the lower reaches of the Yangtze River, Jiangsu Province (see Figure 1). The regions share similar subtropical climates, with average annual temperatures of 18.0 °C and 16.0 °C, where trees, reeds, and weeds are the primary plants, which provide suitable conditions for the survival and reproduction of *O. hupensis*. This area covers approximately 17,000 km^2^, and all snail habitats were included.

### 2.2. Data Collection and Preprocessing

Snail data were obtained from the annual cross-sectional survey conducted by the Jiangsu Institute of Parasitic Diseases. A total of 957 snail study sites were investigated by random sampling method combined with environmental sampling method in March and April from 2017 to 2021 using square lead frames with an area of 0.1 m^2^, placed approximately 10 m apart [26]. The captured snails were crushed and dissected under microscope to observe whether they were alive or dead [27]. The density of *O. hupensis* was calculated according to the number of live snails by the total number of survey frames. The location and survey time of the study sites were also recorded.

Normalized difference vegetation index (NDVI), wetness, and dryness data were extracted from Sentinel-2, which were download from Copernicus Open Access Hub (https://scihub.copernicus.eu/dhus/#/home accessed on 19 August 2021). Land surface temperature (LST) was extracted from Landsat 8 remote sensing (RS) images, which were download from the Geospatial data cloud (https://www.gscloud.cn/ accessed on 19 July 2022). The digital elevation model (DEM) layer and land-use data along the Yangtze River in Jiangsu Province were obtained from Jiangsu Province Surveying and Mapping Engineering Institute, and the slope and aspect of the DEM layer were extracted using ArcGIS10.8 software. The distance to the nearest river (DIS) and population density were extracted from the WorldPop dataset (https://www.worldpop.org/ accessed on 25 August 2021). Soil texture and GDP distribution were downloaded from the Data Center for Resources and Environmental Sciences, Chinese Academy of Sciences (RESDC) (http://www.resdc.cn/ accessed on 11 September 2021). Remote sensing ecological index (RSEI) was calculated as an integrated environment indicator by NDVI, dryness, wetness and LST. Table 1 briefly lists the information of the variables collected in this study, including category, abbreviation, resolution, and source.

1:250,000 scale electronic basic map was downloaded from the national basic geographic database of the National Catalogue Service For Geographic Information (https://www.webmap.cn/main.do?method=index accessed on 15 August 2021), including the administrative boundaries of provinces, counties and water systems. The snail data from 2017 to 2021 were processed in Excel (2019), combined with the explanatory variable data and the longitude and latitude information. These were imported into ArcGIS10.8 and connected to the vector map. The universal horizontal Mercator method was used for projection, and the projection system of all data used WGS 1984 UTM Zone51N as projection coordinates. The Geographical Information System (GIS) database for the snail distribution in the cities studied along the Yangtze River was thus established.

### 2.3. Spatial Autocorrelation

Global and local spatial autocorrelation was used to analyze the spatial distribution of *O. hupensis* at different scales by Moran’s *I* and Getis–Ord Gi* indexes. Value of Moran’s *I* index varies between −1 and 1, with positive values indicating spatial aggregation of observations and negative values indicating that observations tend to be scattered [28]. The greater the absolute value of the index, the greater the correlation of spatial distribution. Gi* index can recognize the spatial clustering of high (hot spot) and low (cold spot) snail density [29]. 

### 2.4. Models Development and Assessment 

In the case of insufficient explanatory variables, the accuracy of the results decreased. Conversely, the modeled results showed a severe multicollinearity problem if all parameters were selected as independent variables [30,31]. Thus, a variance inflation factor (VIF) was introduced to test the collinearity of the independent variables. The diagnostic results demonstrated that the VIF values of variables Wet, Aspect, Soil Texture, Land Use, GDP, and Population, were larger than 7.5 (VIF > 7.5) in the GWR and GTWR models for snail density. We retained the variables NDVI, Dry, LST, RESI, DEM, Slope, and DIS, to calibrate the final GWR and GTWR models based on the standard that VIF should be less than 7.5 [32].

The density of *O. hupensis* was taken as the dependent variable, and the seven environmental factors were elected as the independent variables in the GWR and GTWR models. GWR is expressed as:

(1) $$y_{i}=\beta_{0}\left(u_{i},v_{i}\right)+\overset{p}{\underset{k=1}{\sum }}\beta_{k}\left(u_{i},v_{i}\right)x_{ik}+\varepsilon_{i}\;\;\;\;i=1,2,\cdots ,n,$$

where $y_{i}$ is the snail density in unit i, $\beta_{k}\left(u_{i},v_{i}\right)$ is the kth regression parameter in unit i, $\beta_{0}\left(u_{i},v_{i}\right)$ is the intercept, and $\varepsilon_{i}$ is a random error term.

Compared with the GWR model, which only takes spatial variation into account when estimating an empirical relationship, GTWR captures spatio–temporal heterogeneity based on a weighting matrix referencing both spatial and temporal dimensions. In this study, the Geographical and Temporal Weighted Regression (GTWR) plug-in was installed in ArcGIS, and the GTWR model was created. The basic expression is as follows:

(2) $$y_{i}=\beta_{0}\left(u_{i},v_{i},t_{i}\right)+\overset{K}{\underset{k=1}{\sum }}\beta_{k}\left(u_{i},v_{i},t_{i}\right)X_{ik}+\varepsilon_{i}\;\;\;\;\;\;i=1,2,\cdots ,n,$$

where $y_{i}$ is the snail density in unit i, $\left(u_{i},v_{i},t_{i}\right)$ represents the space–time coordinates of observation i, $\beta_{k}\left(u_{i},v_{i},t_{i}\right)$ is the k regression parameter in unit i, $\beta_{0}\left(u_{i},v_{i},t_{i}\right)$ is the intercept, and $\varepsilon_{i}$ is a random error term. 

In this study, ordinary least squares (OLS), GWR, and GTWR models were compared to explain the relationship between *O. hupensis* and environmental factors, and the optimal model was selected for spatio–temporal visual representation. The adjusted R^2^, Akaike information criterion (AIC), and residual sum of squares (RSS) were determined to evaluate the performance of the models in explaining the density distribution of the snail. The larger the value of the adjusted R^2^ and AIC, the smaller the RSS, the better the model performance. The study takes advantage of Excel for data preprocessing and statistical analysis, ENVI for RS data preprocessing, and ArcGIS 10.8 for spatial analysis, modeling, and mapping. 

## 3. Results

### 3.1. Spatial Distribution of O. hupensis along the Yangtze River in Jiangsu from 2017 to 2021

**Figure 2:**
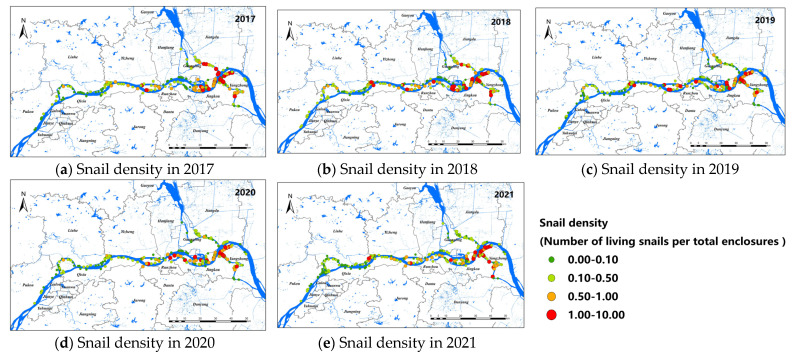
Snail density along the Yangtze River in Nanjing, Zhenjiang, and Yangzhou, 2017–2021 (**a**–**e**).

A total of 957 study sites of *O. hupensis* were selected from 2017 to 2021, including 293 in Nanjing, 439 in Zhenjiang, and 225 in Yangzhou. General speaking, the snail density showed a decreasing trend from 2017 to 2021, as shown in Figure 2. The snail density in Nanjing was relatively low among the three cities, and *O. hupensis* was mainly distributed in Yangzhou and Zhenjiang with a higher density. The study sites of *O. hupensis* at the junction of Nanjing and Yangzhou were relatively higher (the orange and red point in Figure 2).

Moran’s *I* index was used to test the spatial autocorrelation of live snail density. According to the results, Moran’s *I* index of live snail density in the survey region from 2017 to 2021 showed a decreasing trend and then an increasing trend, indicating spatial aggregation (Table 2). During these five years, the *P* values in the overall study area were all less than 0.05, showing a spatial agglomeration trend. In Nanjing, Zhenjiang and Yangzhou, the distribution of *O. hupensis* have statistical differences in spatial distribution, except Nanjing in 2017 and 2019, Zhenjiang in 2019, and Yangzhou in 2019 and 2020.

### 3.2. Hot-Spot Map of Snail Distribution along the Yangtze River in Jiangsu from 2017 to 2021

**Figure 3:**
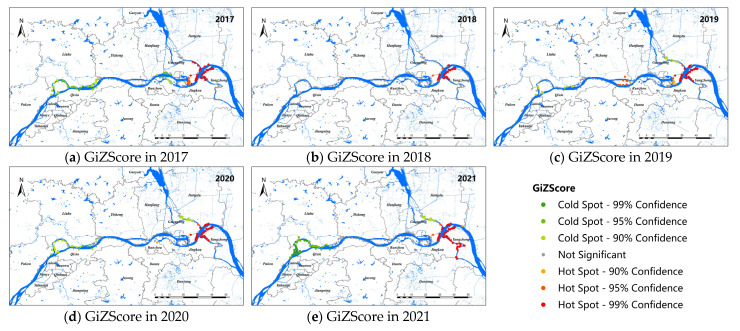
Hot-spot map for snail density along the Yangtze River in Nanjing, Zhenjiang, and Yangzhou, 2017–2021 (**a**–**e**).

Figure 3 shows the hot-spot map of snail distribution in the study area. The results showed that the hot spots of live snail density distributed along the Yangtze River were stable from 2017 to 2021, which were mainly concentrated in the Guangling and Jiangdu districts of Yangzhou City and the Yangzhong and Jingkou districts of Zhenjiang City, and the living snail density was high in the surrounding areas. No obvious cold–cold area was found in 2018. In 2017, the cold–cold spot areas were mainly concentrated in the Luhe, Hanjiang, and Jingkou districts. From 2019 to 2021, the cold-spot density areas were mainly located in the Luhe and Pukou districts of Nanjing, and the junction of the Guangling and Jiangdu districts of Yangzhou. Meanwhile, the number of cold spots had a tendency to increase over the past 3 years, especially in Nanjing. 

### 3.3. Identifying the Determinants of the Distribution of O. hupensis

After collinearity test screening (VIF< 7.5), seven influencing factor variables (NDVI, Dry, LST, RESI, DEM, Slope, and DIS) were used as independent variables to build the model. Pearson’s correlation coefficient between variables and *O. hupensis* density was calculated to explore the strength of association. The results are shown in Table 3, which shows that the correlation between influencing factors and *O. hupensis* density was significantly different. For the whole area, the correlation coefficient of the factors were NDVI (r = 0.082), LST (r = 0.078), DEM (r = 0.069), and DIS (r = −0.069). In Nanjing, NDVI (r = 0.135) and Slope (r = −0.122) were the positive and negative correlation factors, respectively, affecting the density distribution of *O. hupensis*. The correlation coefficients relating to the Zhenjiang area were NDVI (r = 0.161), Dry (r = −0.109), RESI (r = 0.148), and DIS (r = −0.144). In Yangzhou, the positive correlation factor was LST (r = 0.236), and the negative correlation factors were NDVI (r = −0.172) and RESI (r= −0.253).

An OLS regression was performed first, and although the adjusted R^2^ was very low, it provided the basis for subsequent local models. The results (Table 3) showed that the maximum adjusted R^2^ of the OLS model fitting was 0.0229 in the overall group, indicating that 97.71% of snail density was still caused by unknown variables or spatio–temporal heterogeneity.

According to Table 4, in terms of model fitting, the Nanjing, Zhenjiang, and total group snail density GWR models performed best. The GTWR model of *O. hupensis* in the Yangzhou group was significantly better than the OLS and GWR models, and the adjusted R^2^ reached 0.7039, indicating that 70.39% of *O. hupensis* density could be explained. 

Theoretically, on the premise that results of the GTWR model were normal, the standardized residuals should be in a perfect random distribution. The residual of the GTWR model results was diagnosed again with global autocorrelation, as shown in Table 5, and except for the data of the Nanjing group in 2018 (Moran’s *I* = 0.16, *Z* = 2.21, *p* = 0.03), the residual *p* values of the GTWR model were all greater than 0.05, showing no statistical significance, indicating that the model effectively solved the problem of spatial heterogeneity.

### 3.4. Spatial Variation of the Standard Coefficient in GWR

Firstly, the fitting effect of the GWR model was the best according to snail density, in the whole study area (adjusted R^2^ = 0.4481, AICc = 1671.36, RSS = 239.74). The standard coefficients of each variable in the GWR model were counted to clarify the influence of variables on the density of *O. hupensis* (Table 6). The average residual of the model was 0.02, indicating that its accuracy was acceptable. The absolute value of the average standard coefficient was compared as follows: LST (0.75) > DIS (0.64) > Dry (−0.58) > DEM (0.22) > Slope (0.21) > RESI (0.16) > NDVI (0.14). Among them, only Dry had a negative effect on global snail density, and the other factors were positive correlation factors.

**Figure 4:**
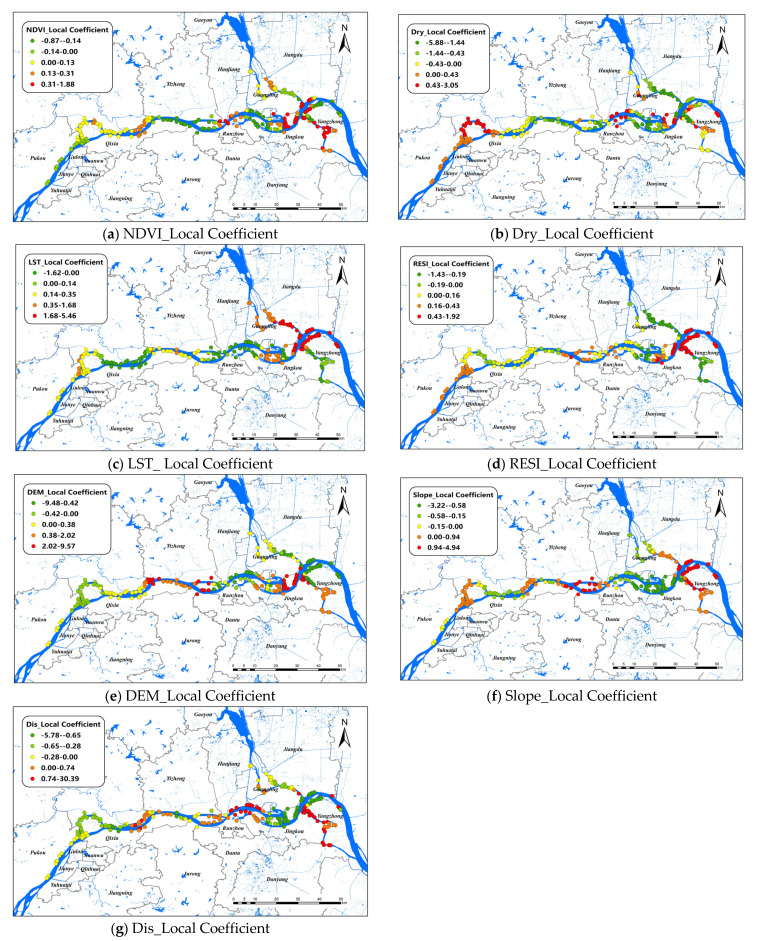
Spatial distribution of impact coefficients of seven factors on snail density in Nanjing, Zhenjiang, and Yangzhou, 2017–2021 (**a**–**g**).

The changes of the explanatory variables of the GWR model in spatial dimensions of *O. hupensis* along the Yangtze River in the three cities during 2017–2021 are mapped in Figure 4. We set “0” as a survey standard for positive and negative effects. The results showed that LST was the most influential environmental factor in this model, and it was positively correlated with snail density in general, especially at the intersection of Guangling, Jiangdu, and Yangzhong districts, whereas the relationship between LST and snail density was inversely proportional in the Luhe and Hanjiang districts. DIS had a positive effect on *O. hupensis* from the lower reaches of the Luhe River to Hanjiang district, as well as a branching channel in Zhenjiang. In other areas, however, the relationship was inversely proportional. Dry was the only factor that had a negative effect on the overall *O. hupensis*, except that the coefficient was greater than zero in the upper reaches of Nanjing and some small parts of Zhenjiang and Yangzhou. Regions with a Slope coefficient greater than 0 were distributed at both ends and in the middle of the study area, while regions with a Slope coefficient less than 0 were located in Luhe, the intersection watershed of the Hanjiang and Jingkou districts. RESI had a negative effect on snail density mainly in the downstream areas, especially in branching channels in Zhenjiang and Yangzhou. The positive effect of NDVI on *O. hupensis* was mainly in the downstream of the study area; however, the effect of NDVI was small, and the correlation was not strong throughout the whole area.

**Figure 5:**
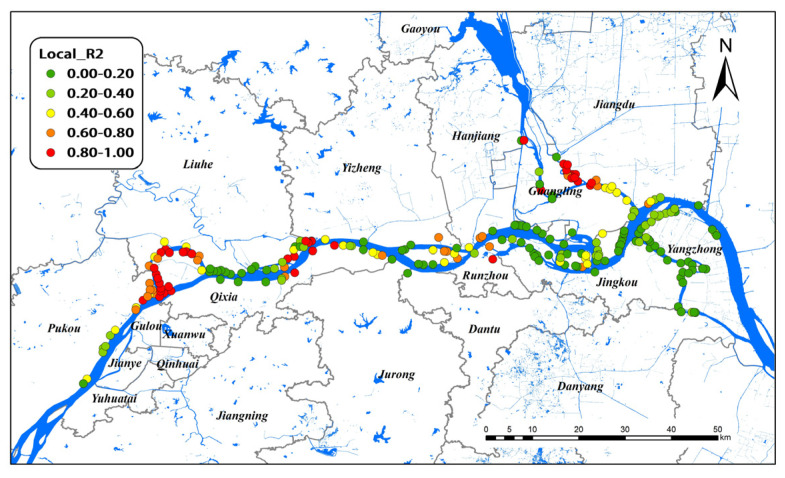
Local R^2^ of GTWR model on snail density in Nanjing, Zhenjiang, and Yangzhou, 2017–2021.

Figure 5 illustrates the spatial distributions of local R^2^ values in the GTWR model for all study areas. In GTWR, branching channels in Nanjing and Yangzhou had very high local R^2^, indicating a decent prediction of the model in these areas. In contrast, the local R^2^ values were lower in the lower reaches of Nanjing and all Zhenjiang areas of the Yangtze River, indicating the poor performance of the model. Considering spatio–temporal heterogeneity, the GTWR model significantly improved the interpretation ability for the Yangzhou group. Therefore, Yangzhou was selected as the research object for spatio–temporal analysis.

### 3.5. Spatial and Temporal Variation of the Standard Coefficient in GTWR

Based on the results of the GTWR model of *O. hupensis* density in Yangzhou, regression coefficients were used to represent the influence of explanatory variables on the dependent variables. The standard coefficients of each variable in the GTWR model were counted to clarify the influence of variables on the density of *O. hupensis* (Table 7). Globally, the average residual of the Yangzhou group model was -0.0039, indicating that the GTWR model had a high accuracy, and the actual snail density was overestimated. The order of the absolute value of the average standard coefficient was similar to that of the GWR model: LST (0.32) > DIS (0.25) > Dry (−0.2) > RESI (−0.17) > DEM (−0.15) > Slope (0.09) > NDVI (−0.02). Among them, LST, DIS, and Slope had positive effects on *O. hupensis* in Yangzhou River beach, and Dry, RESI, DEM, and NDVI were negative factors. 

**Figure 6:**
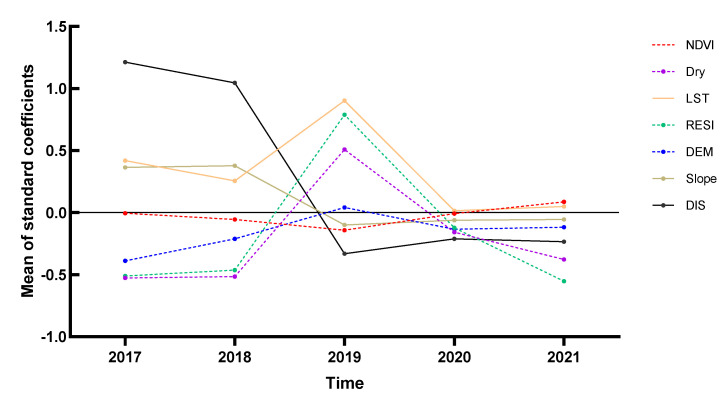
Time series of the standard coefficient of explanatory variables in Yangzhou City, 2017–2021.

The average values of the coefficients between all explanatory variables in Yangzhou City and the snail density in the time dimension are shown in Figure 6, with the folded line representing the effect of different years on snail density for each determinant. From 2017 to 2021, the snail density varied significantly according to the explanatory variables, with a positive correlation of LST, DIS, and Slope. The change trend of the NDVI fit curve was not obvious, and the absolute value was close to 0, indicating that the correlation between NDVI and snail density was not strong. The DIS fit curve showed a U-shaped change and a positive correlation with snail density from 2017 to 2018, and a negative correlation from 2019 to 2021. The fitted curves of Dry, RESI, and DEM showed an inverted V-shape; the top of the inverted V-shape occurred in 2019, and those three explanatory variables were positively correlated with snail density. The LST fit curve revealed a U-shaped change and a positive correlation with *O. hupensis* density. The correlation increased first and then decreased. We found that there was a turning point in 2019 because many of the impact factors fluctuated wildly or even changed sign directions. The LST coefficient increased, and the Dry coefficient changed from negative to positive, indicating that the original breeding sites of *O. hupensis* required a higher surface temperature and dryness. 

**Figure 7:**
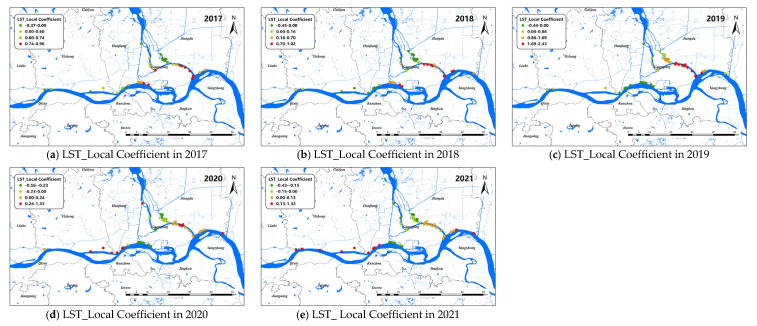
Spatial distribution for impact coefficient of LST factor on snail density in Yangzhou City, 2017–2021 (**a**–**e**).

**Figure 8:**
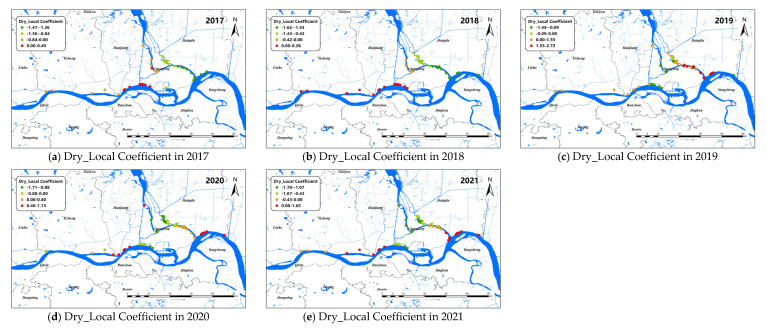
Spatial distribution for impact coefficient of Dry factor on snail density in Yangzhou City, 2017–2021.

The changes of the explanatory variables (e.g., LST and Dry) of the GTWR model in spatial dimensions of *O. hupensis* along the Yangtze River in Yangzhou during 2017–2021 are mapped in Figure 7 and Figure 8. We set “0” as the boundary between positive and negative effects.

LST was positive correlated with snail density on the whole. The areas with high LST coefficients were mainly concentrated in the lower part of the branching channel in Yangzhou, and the absolute value of the LST coefficient decreased over time from 2017 to 2021. In addition, the LST coefficient of the upper reaches of the Yangtze River in Yangzhou City, mainly along the coast of Yizheng City, was also greater than 0 in 2020–2021. The areas with an LST coefficient less than 0 were mainly located in the lower part of the branching channel and Hanjiang district after 2019.

Overall, dryness was negatively correlated with snail density. The Dry coefficients of Yizheng City and Hanjiang district always showed a positive correlation. The Dry coefficient was greater than 0 in Guangling district from 2017 to 2018, indicating the drier the land surface, the higher the snail density. However, after 2019, the Dry coefficient in Guangling district began to show a negative correlation with snail density. Different from Guangling district, the Dry coefficient in Jiangdu district was less than 0 at first, and then showed a positive correlation with snail density after 2019.

## 4. Discussion

This study was designed to explore the spatio–temporal pattern of snail distribution and identify the dynamic determinants of the distribution of *O. hupensis* by GTWR models along the Yangtze River in Jiangsu Province. We firstly analyzed the distribution of snail density in the whole study area and found that the areas with a high density of *O. hupensis* were mainly concentrated at the junction of Zhenjiang and Yangzhou, while there was a trend of cold spots gathering along the Yangtze River in Nanjing, in recent years. Based on RS data, seven influencing factor variables (NDVI, Dry, LST, RESI, DEM, Slope, and DIS) were screened, and a novel *O. hupensis* dataset along the Yangtze River in Jiangsu from 2017 to 2021 was developed by using GWR and GTWR models. Above all, the distribution of the determinants of the whole study area was obtained based on the GWR model. Further, we took Yangzhou as a test area to analyze the dynamic change of the average regression coefficients of each influencing factor in the GTWR model. Clearly, the fluctuation characteristics of environmental factors revealed that the environment had undergone significant annual changes from 2017 to 2021. Thus, different actions can be applied for different environments for the precise prevention and control of *O. hupensis*.

To our knowledge, the reproductive environment of *O. hupensis* serves as the source factor that is responsible for the occurrence, prevalence, and transmission of schistosomiasis, which determine the probability of a regional schistosomiasis epidemic. Among previous studies, a popular research direction has been to analyze the spatial distribution pattern of schistosomiasis to detect the aggregation and aggregation areas of snails and the change of the distribution pattern over time. The geospatial distribution of *O. hupensis* along the Yangtze River in Jiangsu in the current study was similar to those described in previous studies [33,34]. Our results showed that in the process of ecological protection and restoration in the Yangtze River Basin, due to the complex environment, suitable conditions for snail breeding in the beaches along the Yangtze River in Zhenjiang and Yangzhou tended to rebound, and the conditions allowed the snail life cycle to repeat, which indicates the difficulty of snail control. The main reasons for this are as follows. Firstly, the long coastline along the river makes the environment complex, and it is difficult to eliminate the snail. Secondly, *O. hupensis* in the upper reaches of the Yangtze River was not effectively controlled, which led to the snail situations in the lower reaches of the Yangtze River [35]. In addition, due to the limited development along the river, some breeding environments of *O. hupensis* could not be effectively transformed [36]. It should be noted that while monitoring large areas of historical snail environments closely, snails should also be prevented from spreading from the main branch of the Yangtze River to other tributaries. Although the snail density was not the highest in Nanjing along the Yangtze River, it should be noted that there exists a risk of *O. hupensis* diffusion due to flood in 2020 [37], so the surveillance of snails should be continued.

The influencing factors of the distribution of snails in Jiangsu Province agree with the results of other works carried out in marshland, including natural factors such as surface temperature, humidity, soil properties, and vegetation types, as well as human factors such as economic development, population level, and environmental management [38,39]. NDVI and LST are considered to be the most successful environmental factors for predicting snail habitat [40,41]. In this study, LST was the biggest determinant of snail density and was positively correlated with snail density overall. However, the performance of NDVI was not so satisfactory, ranking last among the seven factors. The influence of the distance to nearest river and dryness were second only to LST because one of the important characteristics of *O. hupensis* is concentration in rivers or streams. The flow of water is determined by the elevation of the environment. Therefore, using the DEM to simulate the surface stream network and calculate slope data rapidly and accurately can provide important ecological indexes of *O. hupensis* [12]. However, Jiangsu is flat and consists of plains, waters, low mountains, and hills. Therefore, there is little difference in altitude between the study areas, which may be the reason why DEM and Slope were not as effective as others.

Based on spatial epidemiological methods, domestic and foreign scholars previously carried out spatial heterogeneity research on *O. hupensis* and successfully understood its distribution law at different scales. Jun et al. [38] used a spatial lag model to establish an epidemic risk description method based on land-use type, providing relative estimates of the impact of different land-use types on schistosomiasis prevalence in different regions. Yang et al. [42] utilized a conditional autoregressive model to explore spatial autocorrelation, and combined the data with environmental factors such as LST and vegetation index to construct a Bayesian temporal and spatial model. Yuan et al. [43] used a single-factor logistic regression model to determine the environmental factors related to the distribution of *O. hupensis* in Hubei Province, and then identified the potential high-risk habitats within the spread area of the snail after the flood. These studies have one thing in common: the static influence factors of a time cross-section were selected to predict the distribution of *O. hupensis* or schistosomiasis. However, the development and change of schistosomiasis was a long-term, spatio–temporal, and causal process, and the distribution characteristics, patterns, and trends of *O. hupensis* varied greatly across different regions, times, and socio-economic attributes [44,45].

Geographically and temporally weighted regression is one method to deal with spatial non-stationary data, which can estimate local and global parameters and reflect the spatial effects of factors affecting schistosomiasis. GTWR models have been applied in the modeling of infectious diseases such as hemorrhagic fever of renal syndrome and hand–foot–mouth disease [19,46,47], and they have good modeling accuracy in the study of chronic diseases such as chronic obstructive pulmonary disease [32,48]. After solving the GTWR model, a series of regression coefficients that vary with space and time can be obtained, which can construct a geographical heatmap over time and intuitively predict the spatio–temporal variation amplitude and direction of the determinants on the results. This study verified the feasibility and applicability of this model by fitting snail density along the Yangtze River in Jiangsu Province from 2017 to 2021. 

For the snail density of the whole study area, the GWR model was better than the OLS and GTWR models in fitting, indicating that the dataset had significant spatial variation but no significant temporal non-stationary variation. From the data shown in Figure 4, we found that the factor coefficients were usually of opposite sign in the main and tributaries of the Yangtze River, especially the branching channel in Nanjing. In Luhe district, LST had a positive effect on the upper reaches of *O. hupensis* but the opposite effect on the lower reaches. DIS, Dry, DEM, and other environmental factors were also similar, which were closely related to the Yangtze River flood season. Previous studies found that snail diffusion in the Yangtze River Basin was related to river water velocity, discharge, water level, flooding time, and sediment erosion and deposition [49]. Moreover, during the flood season, the water storage capacity of the tributaries increased, which greatly promoted the spread of *O. hupensis* after the occurrence of flood disaster [50]. The branching channels in Yangzhou and Zhenjiang also experienced a similar phenomenon, which is noteworthy.

The simulation effect of the global GTWR model was inferior to that of the GWR model because the influence factors of different regions differed greatly, which may reduce the estimation ability of the overall data. Figure 5 shows that local R^2^ was relatively high in Nanjing and Yangzhou. The results showed that the GTWR model had a strong ability to explain snail density in Yangzhou. In recent years, the snail area in Yangzhou was at a historically low level, and no positive snails were found. Nevertheless, the phenomenon of reoccurrence often occurred [51]. Snail area in the river beach accounted for the largest proportion, which was where the snails were mainly distributed. Affected by the 2016 Yangtze River flood disaster, the snail situation in Yangzhou rose sharply, and the snail area increased significantly [51]. Beyond these phenomena, the risk of schistosomiasis transmission still exists due to the fairly large water-level changes in the Yangtze River. The snail density in Yangzhou was a turning point in 2019, as many influencing factors fluctuated sharply and even changed their direction. The biggest possibility is that flooding increased the water level and width of the rivers; thus, the snail breeding sites near the river were immersed in water for a longer time, and their density decreased [52]. Although the environmental variables we chose did not consider water level information, this study could indirectly prove that the environmental factors had a substantial impact on the snail distribution from the change trends of Dry and LST.

The Yangtze River Basin is the main area of the schistosomiasis epidemic. It is very important to balance schistosomiasis control while protecting the ecology of the Yangtze River [25]. At present, there is a lack of research on the impact of the Yangtze River restoration project on infectious diseases. By using RS and the GIS, and constructing a GTWR model of the snail growth and decrease in the Yangtze River, we can identify the ecological factors closely related to snail distribution. Therefore, early prevention and control of schistosomiasis in high-risk areas can greatly improve the efficiency of surveillance. RS image data have been widely used to monitor schistosomiasis and the habitats of its intermediate host snails [53,54]. Different from Landsat-8 images with a spatial resolution of 30 m commonly used in previous research, this study extracted environmental indicators from Sentinel-2 images with a spatial resolution of 15 m. In addition, as the terrain of Jiangsu Province is flat and the elevation changes are not obvious, elevation and land use data with a resolution of 2 m were obtained in this study. All these were selected to improve the resolution of explanatory variables so as to improve the accuracy of model fitting. The original breeding sites of *O. hupensis* are often affected by previous snail conditions and snail eradication, which will mask the intensity of spatial and temporal heterogeneity during model construction to some extent. The breeding sites of newly emerging or recurrent *O. hupensis* are largely affected by environmental factors, so the variables screened in this study can have a better explanatory ability.

There are some disadvantages in this study that should be improved. First of all, the spatial resolution of some environmental data in the current study could limit the accuracy of the GTWR models, such as distance to rivers, GDP, and population density. Therefore, it is necessary to carry out further research to analyze the extent to which different scale impact factors can affect the accuracy of prediction results. Another limitation was the representativeness of variables. More factors should be considered to reflect the spatial and temporal characteristics of the snail in GTWR model, e.g., detailed water-level information and the snail-control pesticides used. These factors were not included because the related datasets were difficult to obtain. Moreover, the sampling of snails and environmental elements were conducted only in summer, and, as a result, we often analyzed the obtained data in a cycle unit of one year. In this study, only 5 years of snail data were selected, with the time period being too short to reflect temporal heterogeneity in some areas. Therefore, it is suggested to build a GTWR model on snail data over a longer time axis in the future.

## 5. Conclusions

In this study, the spatial and temporal pattern of *O. hupensis* distribution along the Yangtze River in Jiangsu Province and its dynamic determinants were investigated. The following conclusions can be stated: First, the distribution of *O. hupensis* and the environmental factors in the Jiangsu section of the lower reaches of the Yangtze River had significant spatial aggregation. Second, the determinants affecting the distribution of *O. hupensis* were different in different areas, and, on this basis, environmental transformation for different geographical environmental factors could be used for controlling the snails. Third, the ecological protection and restoration process of the Yangtze River will lead to significant environmental changes, which will affect the distribution of *O. hupensis*. Last, but not least, there is no single scenario that can guarantee the elimination of schistosomiasis in different areas because infection with S. japonicum is epidemiologically distinct throughout its geographical distribution. However, a GTWR model has been prepared and used to identify the dynamic determinants for the distribution of *O. hupensis* and contribute to the national programs of control of schistosomiasis and other snail-borne diseases.

## Figures and Tables

**Table 1:** Explanatory variables used to construct the model in this study, along with descriptions and sources.

Category	Variable Name (Abbreviation)	Resolution	Source
RS	Normalized difference vegetation index (NDVI)	10 m	Sentinal-2
	Wetness (wet)	10 m	Sentinal-2
	Dryness (dry)	10 m	Sentinal-2
	Land surface Temperature (LST)	30 m	Landsat 8
	Elevation (DEM)	2 m	Jiangsu Province Surveying and Mapping Engineering Institute
	Aspect (aspect)	2 m	Calculate by ArcGIS10.8
	Slope (slope)	2 m	Calculate by ArcGIS10.8
	Distance to nearest river (DIS)	1 km	WorldPop
	Remote sensing ecological index (RSEI)	10 m	Sentinal-2
Soil	Soil texture (sand, clay, silt)	1 km	RESDC
Land use	Water, forest, grass, sand beach (land use)	2 m	Jiangsu Province Surveying and Mapping Engineering Institute
Social factors	Distribution of GDP (gdp)	1 km	RESDC
	Density of population (population)	1 km	WorldPop

**Table 2:** Moran’s *I* index and the statistical significance of snail density along the Yangtze River in Nanjing, Zhenjiang, and Yangzhou, 2017–2021.

Year	All			Nanjing			Zhenjiang			Yangzhou		
	Moran’s *I*	*Z*	*p*	Moran’s *I*	*Z*	*p*	Moran’s *I*	*Z*	*p*	Moran’s *I*	*Z*	*p*
2017	0.39	10.32	0.00	0.18	1.82	0.07	0.29	3.57	0.00	0.41	4.34	0.00
2018	0.25	4.42	0.00	0.68	9.76	0.00	0.16	2.14	0.03	0.32	2.15	0.03
2019	0.24	5.45	0.00	0.06	1.14	0.26	0.15	1.89	0.06	0.08	0.83	0.40
2020	0.29	6.65	0.00	0.38	5.31	0.00	0.19	2.34	0.02	0.20	1.63	0.10
2021	0.32	8.66	0.00	0.15	4.20	0.00	0.23	2.84	0.00	0.30	2.29	0.02

**Table 3:** Pearson correlation coefficients for potential influencing factors and *O. hupensis* density.

Affecting factors	Correlation coefficient			
	All	Nanjing	Zhenjiang	Yangzhou
NDVI	0.082 *	0.135 *	0.161 **	−0.172 **
Dry	−0.038	−0.053	−0.109 *	0.065
LST	0.078 ^*^	0.081	0.015	0.236 **
RESI	0.041	0.103	0.148 **	−0.253 **
DEM	0.069 *	−0.015	−0.037	−0.045
Slope	−0.009	−0.122 *	0.069	−0.079
DIS	−0.069 *	0.107	−0.144 **	0.045

* *p* < 0.05, ** *p* < 0.01.

**Table 4:** Evaluating the performance of the OLS, GWR, and GTWR models.

Group	Model	R^2^	Adjusted R^2^	AICc	RSS
All	OLS	0.0300	0.0229	1955.08	424.05
(n = 957)	GWR	0.4522	0.4481	1671.36	239.74
	GTWR	0.3373	0.3324	1814.36	290.03
Nanjing	OLS	0.0459	0.0224	78.18	85.82
(n = 293)	GWR	0.5083	0.4962	64.30	11.16
	GTWR	0.4597	0.4464	60.93	12.26
Zhenjiang	OLS	0.0716	0.0565	1102.40	303.64
(n = 439)	GWR	0.4365	0.4273	1017.71	184.72
	GTWR	0.3504	0.3398	1046.87	212.94
Yangzhou	OLS	0.1352	0.1073	117.75	20.45
(n = 225)	GWR	0.6062	0.5935	47.44	9.35
	GTWR	0.7132	0.7039	29.10	6.81

**Table 5:** Moran’s *I* index and statistical significance of GTWR residual in Nanjing, Zhenjiang, and Yangzhou, 2017–2021.

Year	All			Nanjing			Zhenjiang			Yangzhou		
	Moran’s *I*	*Z*	*p*	Moran’s *I*	*Z*	*p*	Moran’s *I*	*Z*	*p*	Moran’s *I*	*Z*	*p*
2017	0.01	0.79	0.43	−0.15	−1.49	0.14	0.10	1.88	0.06	−0.06	−0.50	0.62
2018	−0.06	−1.83	0.07	0.16	2.21	0.03	−0.07	−1.15	0.25	−0.03	−0.13	0.90
2019	−0.04	−1.59	0.11	−0.08	−1.26	0.21	−0.05	−0.63	0.53	−0.02	0.12	0.90
2020	0.02	0.88	0.38	−0.06	−0.81	0.42	−0.01	0.01	0.99	−0.04	−0.29	0.77
2021	−0.01	−0.02	0.97	−0.04	−1.08	0.28	−0.01	0.00	0.99	−0.01	0.31	0.75

**Table 6:** Statistical description of GWR of snails in Nanjing, Zhenjiang, and Yangzhou, 2017–2021.

Variable	Mean	Std. Dev	Minimum	Maximum
Intercept	−0.06	0.86	−4.21	1.68
NDVI	0.14	0.47	−0.88	1.88
Dry	−0.58	1.57	−5.88	3.05
LST	0.75	1.28	−1.62	5.46
RESI	0.16	0.55	−1.42	1.92
DEM	0.22	2.84	−9.48	9.57
Slope	0.21	1.39	−3.21	4.94
DIS	0.64	4.60	−5.78	30.39
Residual	−0.02	0.50	−1.47	6.28
R^2^	0.4522			

**Table 7:** Statistical description of GTWR of snails in Yangzhou City.

Variable	Mean	Std. Dev	Minimum	Maximum
Intercept	0.33	1.27	−4.07	2.51
NDVI	−0.02	0.25	−0.64	0.33
Dry	−0.20	0.91	−1.71	2.72
LST	0.32	0.64	−0.56	2.42
RESI	−0.17	1.19	−2.40	4.12
DEM	−0.15	0.24	−0.90	0.28
Slope	0.09	0.46	−0.53	1.59
DIS	0.25	1.05	−0.95	3.00
Residual	0.00	0.17	−0.40	0.97
R^2^	0.7132			

## Data Availability

Third party data, not applicable.
